# A PDMS-Based Cylindrical Hybrid Lens for Enhanced Fluorescence Detection in Microfluidic Systems

**DOI:** 10.3390/s140202967

**Published:** 2014-02-13

**Authors:** Bor-Shyh Lin, Yu-Ching Yang, Chong-Yi Ho, Han-Yu Yang, Hsiang-Yu Wang

**Affiliations:** 1 Institute of Imaging and Biomedical Photonics, National Chiao Tung University, Tainan 71150, Taiwan; E-Mails: borshyhlin@mail.nctu.edu.tw (B.-S.L.); joviy@unice.com.tw (Y.-C.Y.); harryspiderman@gmail.com (H.-Y.Y.); 2 Department of Medical Research, Chi-Mei Medical Center, Tainan 71004, Taiwan; 3 Department of Chemical Engineering, National Cheng Kung University, Tainan 70101, Taiwan; E-Mail: hochongyi@gmail.com

**Keywords:** microfluidic systems, optical components, hybrid lens, fluorescence measurement, polydimethylsiloxane (PDMS)

## Abstract

Microfluidic systems based on fluorescence detection have been developed and applied for many biological and chemical applications. Because of the tiny amount of sample in the system; the induced fluorescence can be weak. Therefore, most microfluidic systems deploy multiple optical components or sophisticated equipment to enhance the efficiency of fluorescence detection. However, these strategies encounter common issues of complex manufacturing processes and high costs. In this study; a miniature, cylindrical and hybrid lens made of polydimethylsiloxane (PDMS) to improve the fluorescence detection in microfluidic systems is proposed. The hybrid lens integrates a laser focusing lens and a fluorescence collecting lens to achieve dual functions and simplify optical setup. Moreover, PDMS has advantages of low-cost and straightforward fabrication compared with conventional optical components. The performance of the proposed lens is first examined with two fluorescent dyes and the results show that the lens provides satisfactory enhancement for fluorescence detection of Rhodamine 6G and Nile Red. The overall increments in collected fluorescence signal and detection sensitivity are more than 220% of those without lens, and the detection limits of Rhodamine 6G and Nile red are lowered to 0.01 μg/mL and 0.05 μg/mL, respectively. The hybrid lens is further applied to the detection of Nile red-labeled *Chlorella vulgaris* cells and it increases both signal intensity and detection sensitivity by more than 520%. The proposed hybrid lens also dramatically reduces the variation in detected signal caused by the deviation in incident angle of excitation light.

## 1. Introduction

Fluorescence detection has been widely used in biological and chemical applications, such as DNA quantifications and cell classification [1,2]. In order to build a portable fluorescence detecting system, miniature systems with multiple integrated functions to perform a complete series of biochemical analysis have been proposed and implemented [3–9]. Several research teams have applied light source and detector with color filters or polarizer directly to microchannels [10–12]. In order to collect more fluorescence, they attempted to enhance the intensity of the excitation light, but the incidence angle and the transmission path of the excitation light are difficult to control because the light source is divergent. Furthermore, compared with the intensity of the excitation light, the intensity of the collected fluorescence is extremely weak. This greatly increases the detection limit in fluorescence measurements.

The use of optical fibers was also proposed to guide light with accuracy and low loss [13,14]. The loss of light transmission can be effectively reduced by the total reflection in fiber tubes and their flexible characteristics can also enable close arrangement between the fiber and the detector. However, the main drawback is the weak coupling between light sources, microchannel, and detectors, which causes the loss of excitation light or fluorescence. Some microfluidic chips have embedded waveguides near the front tip of the embedded optical fiber to guide light close to the microchannel to reduce the loss of excitation light or fluorescence [15–17], but these on-chip optical components require high-quality fabrication and the optical arrangement is not flexible after the fabrication. Limiting the detection area to a specific region can result in considerable errors, especially when the sample is heterogeneous or non-uniform. Moreover, most of these on-chip optical components focus on effective delivery of excitation light to the sample. Microscopes or other equipment with high optical sensitivity are required for the detection of the weak emission light. Therefore, a straightforward, low-cost, and flexible setup is required to build an economically affordable and high performance detection system, which provides not only focused excitation but also enhanced fluorescence collection.

In this study, a miniature, cylindrical, and hybrid lens made of PDMS is proposed to improve the efficiency of excitation light delivery and fluorescence collection in microfluidic systems. The proposed hybrid lens combines a laser focusing lens and a fluorescence-collecting lens to focus the incident laser onto the interactive region of microfluidic channel and collect the isotropic fluorescence simultaneously. The autofluorescence of PDMS is low and comparable to that of Borofloat^®^ glass, making it a great substitute for the conventional lens material [18]. Moreover, the advantages of flexible design and straightforward fabrication make our PDMS hybrid lens more promising compared with conventional bulk optics in facilitating the commercialization of economically viable point-of-care systems based on optical detection. The proposed hybrid lens was put into use and its performance is validated using two common fluorescent probes, Rhodamine 6G and Nile Red, as well as Nile Red- labeled cells of the microalgae *Chlorella vulgaris*.

## 2. Hybrid Lens Design

**Figure 1.**
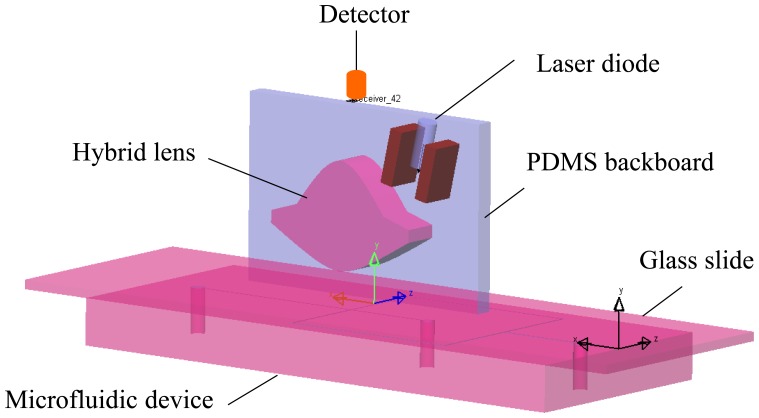
System scheme of PDMS-based cylindrical hybrid lens used in microfluidics.

**Figure 2.**
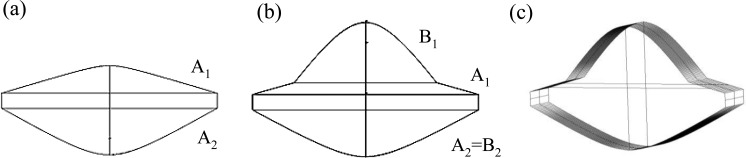
Schematics of: (**a**) Laser focusing lens; (**b**) Hybrid lens combining fluorescence collecting lens (B_1_ + A_2_) and laser focusing lens (A_1_ + A_2_); (**c**) Cylindrical hybrid lens with functions of laser focusing and fluorescence collecting.

The optical system with the proposed miniature hybrid cylindrical lens for laser focusing and fluorescence collecting is shown in Figure 1. The two lenses are integrated on a PDMS backboard. The incident laser enters the hybrid lens and then is focused just below the glass slide. The microfluidic device is positioned below the collecting lens with the glass slide facing the lens. A spectrometer is placed at the focal point of the collecting lens. Focusing the excitation light is beneficial for enhancing the induced fluorescence. Additionally, it also increases the signal to noise ratio (SNR) by reducing the effects from strayed or environmental light. The more efficient the focusing is, the more fluorescence could be induced and detected. The dimensions and characteristic parameters of the laser focusing lens, the fluorescence collecting lens, and the hybrid lens are shown in Figure 2 and Table 1.

The laser focusing lens in Figure 2a is designed to focus the 532 nm incident laser on the floor of the microchannel, which is immediately next to the glass slide. Compared with the excitation light, the induced fluorescence is much weaker and emits in an isotropic direction, resulting in the loss of a large proportion of the original intensity when detecting with a small detector. Therefore, it is necessary to design another lens to collect fluorescence. Because the laser beam passes through only a little part of surface A_1_ of the laser focusing lens, a fluorescence collecting lens is added at the center of surface A_1_ for fluorescence collecting, and the resulting hybrid lens is shown in Figure 2b. The fluorescence collecting lens also uses surface A_2_ in the laser focusing lens for light collection. Therefore, the hybrid lens has dual functions: the incident laser is focused by surface A_1_ and A_2_ and fluorescence is collected by surface A_2_ and B_1_. Because aspheric lenses are expensive to manufacture, the hybrid lens is designed with a cylindrical structure in this study, as shown in Figure 2c. Although the cylindrical structure may have lower performance compared with spherical ones, it is easier and cheaper to manufacture and it provides the desired enhancement for fluorescence detection. The width of the hybrid lens is 18.36 mm and the height is 11.84 mm. This hybrid lens is applied in all experiments in this study.

## 3. Materials and Methods

### 3.1. Hybrid Lens Fabrication

**Figure 3.**
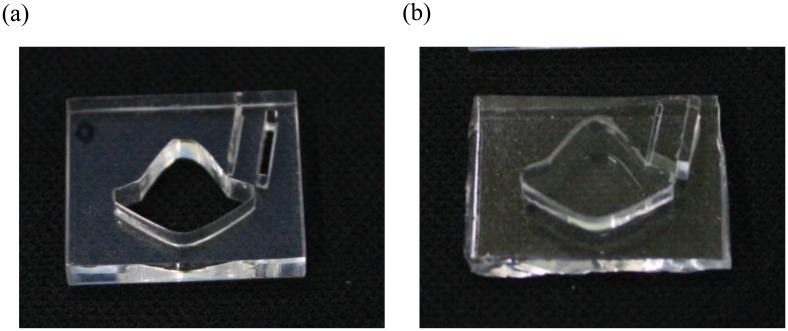
(**a**) PMMA mold and (**b**) PDMS hybrid lens.

The polymethylmethacrylate (PMMA) mold for casting the hybrid lens is made by a CNC laser cutting machine (Mcl Recruitment, Blackburn, UK). PMMA is chosen because it is easy to modify and has a relatively smooth surface. Polydimethylsiloxane (PDMS, RTV 615, Momentive, Waterford, NY, USA) is used as the material to replicate the hybrid lens. A desired amount of mixed PDMS solution (A:B = 10:1) is poured over the mold and vacuum is applied for 20 min to remove bubbles in the mixture. The PDMS mixture is then cured in the oven at 150 °C for 10 min and the cured sheet is peeled off of the mold. Figure 3a,b shows the PMMA mold and the replicated PMDS hybrid lens, respectively.

### 3.2. Fluorescence Measurement Setup

**Figure 4.**
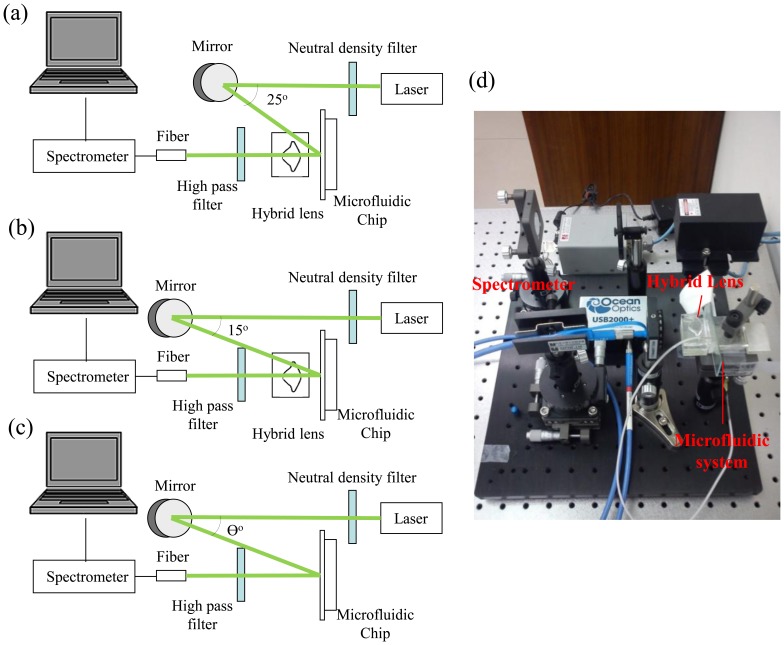
Optical setups of (**a**) measurement using fluorescence collecting lens; (**b**) measurement using hybrid lens; and (**c**) measurement without lens, θ is 15° or 25°, depending on experiments; (**d**) Photography of the optical experiment setup in (**b**).

The optical measurement setup is shown in Figure 4. A 532 nm laser is aligned to a reflected mirror to adjust laser incident angle. The hybrid lens and microfluidic device are mounted on a platform. A spectrometer (USB2000+, Ocean Optics, Dunedin, FL, USA) connected with a 400 um multimode fiber (QP400-2-VIS-NIR, Ocean Optics) works as the sensor for detecting fluorescence intensity. In order to enhance the quality of the collected fluorescence, a 550 nm filter (FEL0550, Thorlabs, Newton, NJ, USA) is placed between the fiber and hybrid lens to cut off the scattered 532 nm laser light.

Figure 4a shows the setup of fluorescence collecting lens measurement. In order to enhance the collection efficiency of the fluorescence collecting lens, the laser beam is incident on the microfluidic chip at 25° without passing the laser focusing lens. Figure 4b shows the measurement setup using the hybrid lens. The laser beam is incident on the hybrid lens at 15°. The fluorescence intensity detected without PDMS lens as shown in Figure 4c was acquired in each set of experiments to compensate the variation caused by the difference in incident angles. A photography of the optical experiment setup is shown in Figure 4d.

### 3.3. Microfluidic Device

The microfluidic device was fabricated using standard soft lithography as detailed in our previous work [19]. The pattern of the microfluidic channel was drawn using auto-CAD software and printed on a transparency. The microfluidic pattern was then transferred to a silicon wafer by lithography. A silicon wafer was then utilized as the template for replicating PDMS sheets containing a straight microchannel (height: 125 μm, width: 50 μm). The PDMS sheet was treated with oxygen plasma before sealing onto a glass slide to form the closed channel. Connection holes between the microfluidic channel and tubings were punched with a blunt end needle. Fluorescent solutions containing different concentrations of Rhodamine 6G or Nile Red (Sigma-Aldrich Co. LLC., St. Louis, MO, USA) in DMSO were injected into the channel using a syringe pump (KD Scientific Inc., Holliston, MA, USA). The cultivation conditions for *Chlorella vulgaris* culture can be found in our previous report [20]. The cells were obtained from the culture tank and adjusted to different optical densities (O. D.) for the fluorescence detection. After labelled with 0.08 M Nile Red, the cells were centrifuged to remove excessive dye and resuspended in phosphate buffer (8.125 g KH_2_PO_4_ in 500 mL of deionized water) before infusing into the microchannel.

## 4. Results and Discussion

**Figure 5.**
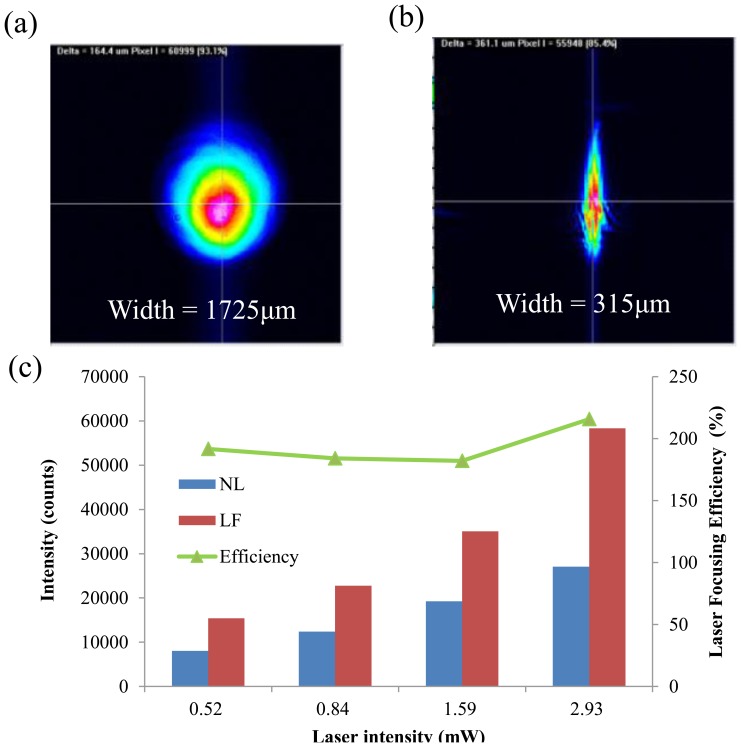
Beam profile images (**a**) without lens (NL); and (**b**) with laser focusing lens (LF); (**c**) Intensities of original and focused laser beam, and the focusing efficiency of the proposed lens.

In order to evaluate the performance of the proposed hybrid lens for laser focusing, a laser beam profiler (BEAMAGE-CCD12, Gentec-eo Inc., Quebec, QC, Canada) is used to measure the beam properties. Figure 5a and Figure 5b show the profiles of the original laser beam and the focused laser beam after passing through the proposed hybrid lens, respectively. They show that the width of the focused laser beam (315 μm) is significantly narrower than that of the original laser beam (1,725 μm). The laser focusing lens can thus provide an 81.7% reduction of spot size. The intensities of the original laser and the focused laser are shown in Figure 5c. Here, the focusing efficiency is defined as follows:

(1) $$\text{Focusing efficiency}(\%)=\frac{{\text{I}}_{\text{with lens}}}{{\text{I}}_{\text{withoutlens}}}\times 100\%$$

where *I_withlens_* and *I_withoutlens_* denote the light intensity after passing the hybrid lens and without the hybrid lens, respectively. The fluorescence collection efficiency for the fluorescence-collecting lens and the overall efficiency for hybrid lens are also defined using the same principle. The result shows that the intensity of the focused laser increased to 194% compared with the original beam. Therefore, the laser focusing lens certainly enhances the intensity of the excitation light. Additionally, no significant increase in laser intensity noise was observed after applying the PDMS lens.

**Figure 6.**
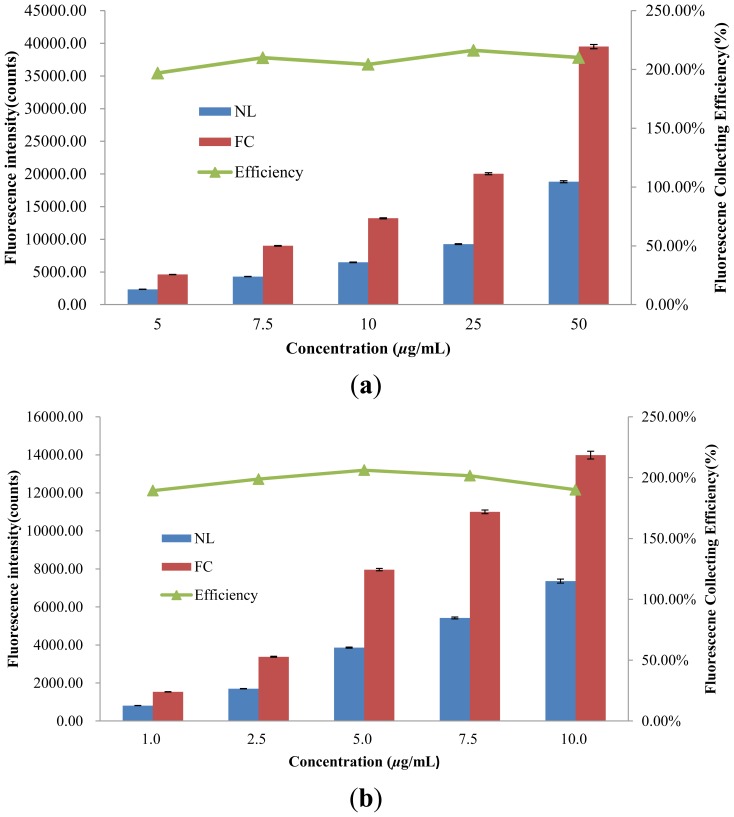
The fluorescence intensity obtained with fluorescence collecting lens (FC) and without lens (NL). The efficiency for fluorescence collection is also plotted for different concentrations of (**a**) Nile red and (**b**) Rhodamine 6G. The excitation laser has an incident angle of 25°.

The fluorescence-collecting efficiency of the proposed lens for fluorescence measurement in the microfluidic channel is investigated using the setup in Figure 4a. Figure 6a,b is the intensities of the collected fluorescence at different concentrations of Nile Red and Rhodamine 6G (R6G), respectively. For Nile Red concentrations ranging from 5 to 50 μg/mL, the attached spectrometer, as shown in Figure 4c, can collect fluorescence counts from 2,340 to 18,800. When the lens is applied, the collected fluorescence counts increase from 4,607 to 39,505, indicating that the fluorescence-collecting lens has a collecting efficiency around 200%. Applying the fluorescence-collecting lens to the detection of Rhodamine 6G gives a similar enhancement. In additional to the stable collecting efficiency, the fluorescence-collecting lens also increases the sensitivity of the detection to two fold for both fluorescent dyes (Figure S1). The correlation between signal intensity and dye concentration remains linear when the fluorescence-collecting lens is applied; therefore, quantitative analysis is feasible and straightforward.

**Figure 7.**
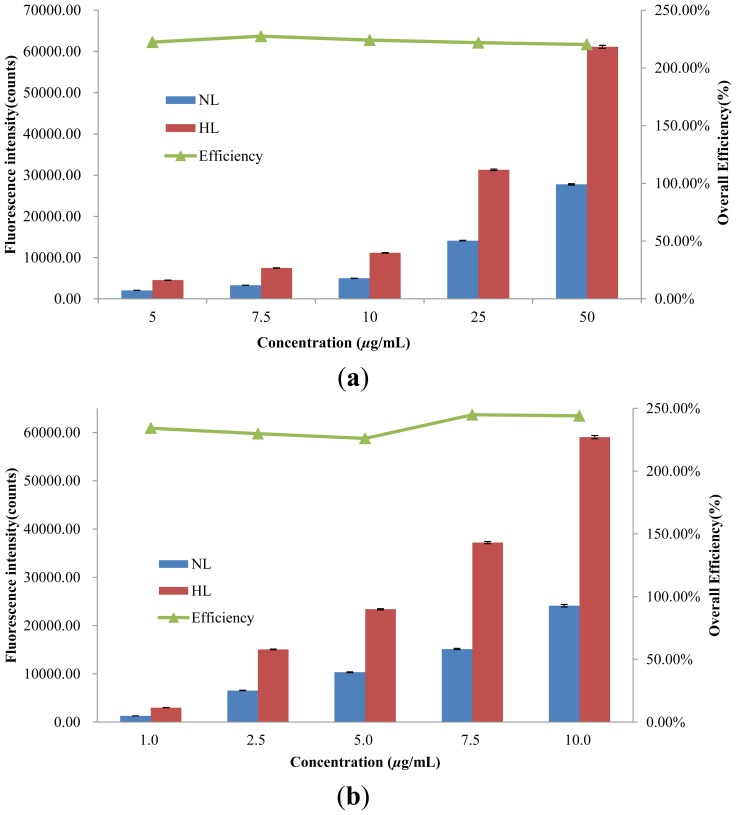
The fluorescence intensity obtained with hybrid lens (HL) and without lens (NL). The overall efficiency in fluorescence detection is also plotted for different concentrations of (**a**) Nile red and (**b**) Rhodamine 6G. The excitation laser has an incident angle of 15°.

The deployment of the hybrid lens as shown in Figure 4b further increases the detected fluorescence intensity and detection sensitivity. As shown in Figure 7a, the detected fluorescence intensities without the hybrid lens range from 2,031 to 27,678 when the Nile Red concentration increases from 5 to 50 μg/mL. When hybrid lens is applied, the detected fluorescence intensity increases from 4,515 to 60,991 in the same concentration range.

**Figure 8.**
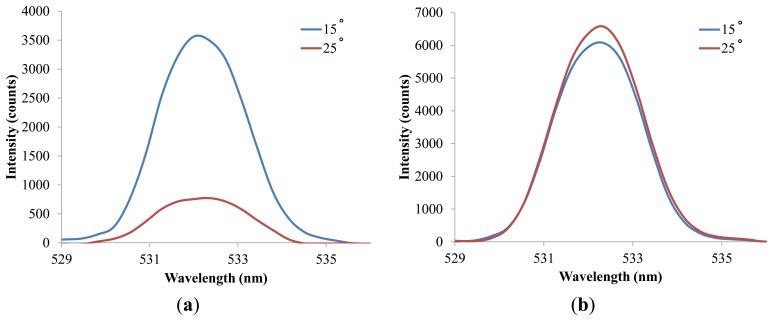
The collected light intensity at different incident angles for excitation light when (**a**) no lens is applied; (**b**) the hybrid lens is applied.

A similar trend is observed in the experiments with Rhodamine 6G. The overall efficiency of the hybrid lens is around 225% and 235% for Nile red and Rhodamine 6G, respectively, while the corresponding enhancements in sensitivity are around 220% and 250% (Figure S2). The differences in the overall efficiencies and sensitivities for Nile Red and Rhodamine 6G can be attributed to the broad emission band of Nile Red and the nature of the fluorescence intensity quantification method used in this study. The peak intensity is applied to calculate the enhancement in fluorescence detection and this can result in underestimation of emission signals when the excited dye molecules release energy via light of various wavelengths. However, quantifying peak intensity or fluorescence intensity at a fixed wavelength is a more straightforward strategy when it comes to the construction of portable optical systems or point of care systems since it only requires a simple and inexpensive photodiode as the detector. It is also noticed that the overall efficiency of the hybrid lens is lower than the expected value, which is the product of focusing efficiency and fluorescence efficiency. This reduction arises from the difference in incidence angles in the fluorescence detection using the fluorescence-collecting lens and hybrid lens. Figure 8a shows the effect of incidence angle on the detected light intensity without lens and an incidence angle of 15°, which is applied in the hybrid lens examination, results in a significantly higher signal. However, when the lens is applied, the difference in incidence angle only produces an 8% variation in the detected light intensity as shown in Figure 8b. Consequently, the overall efficiency is lower than the value predicted by the data obtained from the fluorescence-collecting lens examination in which a 25° incidence angle is applied. Figure 8 also shows the advantage of using the proposed lens to perform fluorescence detection. Since the signal is less sensitive to the laser incidence angle when the lens is applied, the alignment demands are reduced and consequently, experimental errors due to misalignment can be brought down.

**Figure 9.**
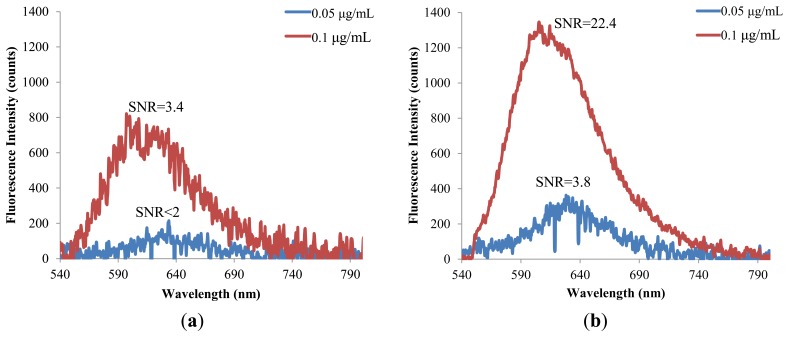
The detection limits of Nile red: (**a**) without lens and (**b**) with hybrid lens.

**Figure 10.**
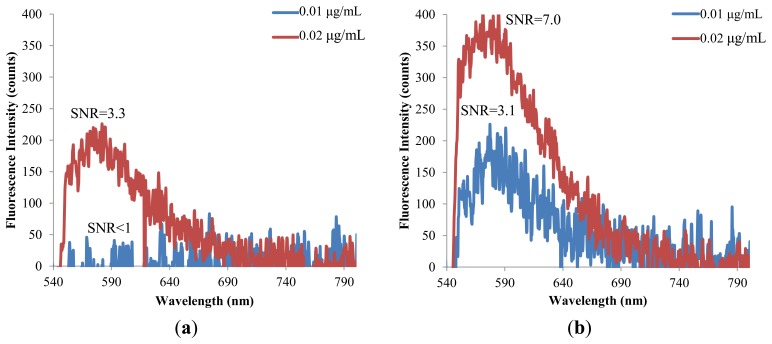
The detection limits of Rhodamine 6G (**a**) without lens and (**b**) with hybrid lens.

The hybrid lens is further applied to examine the detection limit for the fluorescent molecules. A reading is recognized as a meaningful signal in this study when the signal to noise ratio (SNR) is higher than 3. Figure 9 shows that the detection limit of Nile Red is lowered from 0.1 to 0.05 μg/mL when the hybrid lens is applied. Similarly, the detection limit of Rhodamine 6G is lowered to half of the original value by the hybrid lens, as shown in Figure 10.

These detection limits for fluorescent molecules are much lower than those reported in a previous study using a PDMS lens [21]. These data demonstrate the feasibility of using the proposed PDMS hybrid lens to construct low cost optical systems with satisfactory performance. It should be noted that the proposed PDMS lens is replicated from an untreated PMMA mold and the surface can be much rougher than ideal. If a suitable surface smoothing strategy such as solvent vapor treatment [22] were applied, the performance of the proposed lens could be further improved.

**Figure 11.**
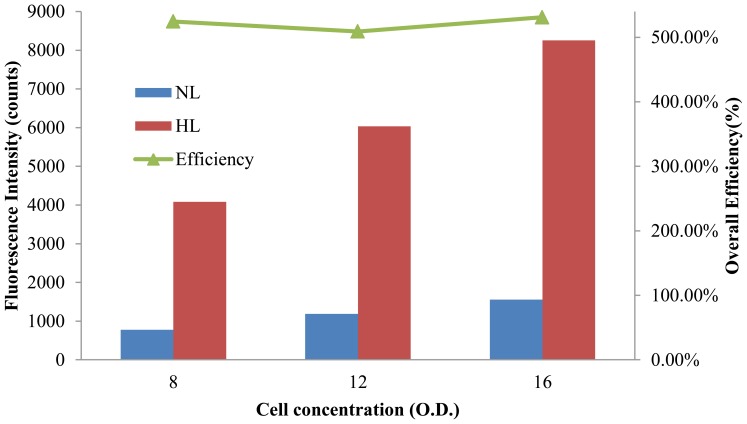
The fluorescence intensity obtained with the hybrid lens (HL) and without lens (NL) in the detection of Nile Red-labeled *Chlorella vulgaris* cells. The overall fluorescence detection efficiency is also plotted for different concentrations (O.D.) of cells.

In addition to dye solutions, the performance of the hybrid lens for cell detection was also examined with Nile Red-labeled *Chlorella vulgaris* cells and the results are shown in Figure 11. Nile Red is often applied to visualize cellular lipids inside microalgae for the screening of cell strains suitable for mass production of biodiesel [23]. Since the light emitted by cells is much lower than that from dye solutions, the integration time was extended to 3 sec to obtain decent signals in the experiments without the hybrid lens. Similar to the detection of fluorescent dyes, the signal intensity increased linearly with the sample concentration (O.D.). The efficiency of the hybrid lens for the detection of labeled *Chlorella vulgaris* cells was around 520% and the detection sensitivity was increased by 540%. It should also be noted that the long integration time barely increased the detection background. These data indicate that the hybrid lens has great potential for applications in biomedical detections in which the cell amounts are limited.

A summary of the efficiency of the proposed hybrid lens for Nile Red and R6G is shown in Table 2. For dye solutions, the proposed hybrid lens produces an overall fluorescence detection enhancement of 235%, which is comparable to the previously reported PDMS 2D-optical lens design [21]. However, the PDMS lens in [21] is fabricated as an on-chip component and can only detect samples in specific positions. Moreover, the proposed hybrid lens has a much higher efficiency, 520%, in the fluorescence detection of *Chlorella vulgaris* cells. The lens proposed in this study is not coupled with the microchannel and was designed with the purpose for constructing portable and flexible detection instruments. Furthermore, the proposed lens reduces the alignment demands of optical components and enhances the accuracy and reproducibility of fluorescence detection. The straightforward fabrication and inexpensive substrate of the PDMS lens also facilitates the implementation of economically viable optical systems with adequate performance.

## 5. Conclusions

In this study, a miniature, cylindrical, hybrid PDMS-based lens is proposed to improve the quality of fluorescence detection in microfluidic systems. The proposed hybrid lens can focus the excitation light and increase the efficiency of fluorescence collection simultaneously. The performance of the PDMS-based hybrid lens is validated using two common fluorescent dyes and *Chlorella vulgaris* cells labeled with Nile Red. The experimental results show that the laser focusing lens can provide an 81.7% reduction of spot size and 194% enhancement for excitation light. For fluorescent dyes, the fluorescence-collecting lens increases the detected signal by 200% for both Rhodamine 6G and Nile Red. The overall efficiency of the hybrid lens for the dyes can be as high as 235% and the detection limits of Nile red and Rhodamine 6G are reduced to 0.05 μg/mL and 0.01 μg/mL, respectively. Furthermore, the hybrid lens enhances the signal intensity and detection sensitivity of *Chlorella vulgaris* cells by more than 520%. The use of the hybrid lens also alleviates the variation of signal caused by component misalignment and increases the sensitivity of detection by more than two folds. The proposed hybrid lens provides a straightforward and effective way to enhance the quality of fluorescence detection for the tiny amount of sample in microfluidic devices. It has great potential in building portable and economically viable point-of-care testing systems based on fluorescence detection.

## Figures and Tables

**Table 1.** Parameters of the laser focusing lens and fluorescence collecting lens.

**Parameter**	**Laser Focusing Lens**		**Fluorescence Collecting Lens**	
	
	**A_1_**	**A_2_**	**B_1_**	**B_2_**
Inner diameter (mm)	18.36	18.36	16.32	18.36
Conic constant	−11.08	−3.58	−1.4	−3.58
Curvature	0.21	0.22	0.5	0.22
Focal length (mm)	7.55	7.55	4.93	11.31

**Table 2.** Summary of efficiency of proposed lens for Nile Red and R6G (FC: Fluorescence collecting lens; HL: Hybrid lens).

**Dye**	**Conc. of Dye (μg/mL)**	**Efficiency of FC (%)**	**Efficiency of HL (%)**
**Nile Red**	5	197	222
	7.5	210	228
	10	204	224
	25	216	222
	50	210	220

**R6G**	1	189	234
	2.5	199	230
	5	206	226
	7.5	203	245
	10	190	244
