# Supplementary material for: A PDMS-Based Cylindrical Hybrid Lens for Enhanced Fluorescence Detection in Microfluidic Systems
Source: Sensors (Basel). 2014 Feb 13;14(2):2967–80. doi: 10.3390/s140202967 (PMC3958222; doi:10.3390/s140202967)
Supplement: Supplementary file 1 [file sensors-14-02967-s001.pdf]

OPEN ACCESS
sensors
ISSN 1424-8220
www.mdpi.com/journal/sensors

Supplementary Information

A PDMS-Based Cylindrical Hybrid Lens for Enhanced Fluorescence Detection in Microfluidic Systems. *Sensors* 2014, *14*, 2967-2980

Bor-Shyh Lin [1,2], Yu-Ching Yang [1], Chong-Yi Ho [3], Han-Yu Yang [1] and Hsiang-Yu Wang [3,*]

[1] Institute of Imaging and Biomedical Photonics, National Chiao Tung University, Tainan 71150, Taiwan; E-Mails: borshyhlin@mail.nctu.edu.tw (B.-S.L.); joviy@unice.com.tw (Y.-C.Y.); harryspiderman@gmail.com (H.-Y.Y.)
[2] Department of Medical Research, Chi-Mei Medical Center, Tainan 71004, Taiwan
[3] Department of Chemical Engineering, National Cheng Kung University, Tainan 70101, Taiwan; E-Mail: hochongyi@gmail.com

* Author to whom correspondence should be addressed; E-Mail: hywang@mail.ncku.edu.tw; Tel.: +886-6-27-57575 (ext. 62648).

Figure S1. The sensitivity (slope) of the fluorescence detections with fluorescence collecting lens (FC) and without lens (NL) for (**a**) Nile red and (**b**) Rhodamine 6G. The excitation laser has an incident angle of 25 °.

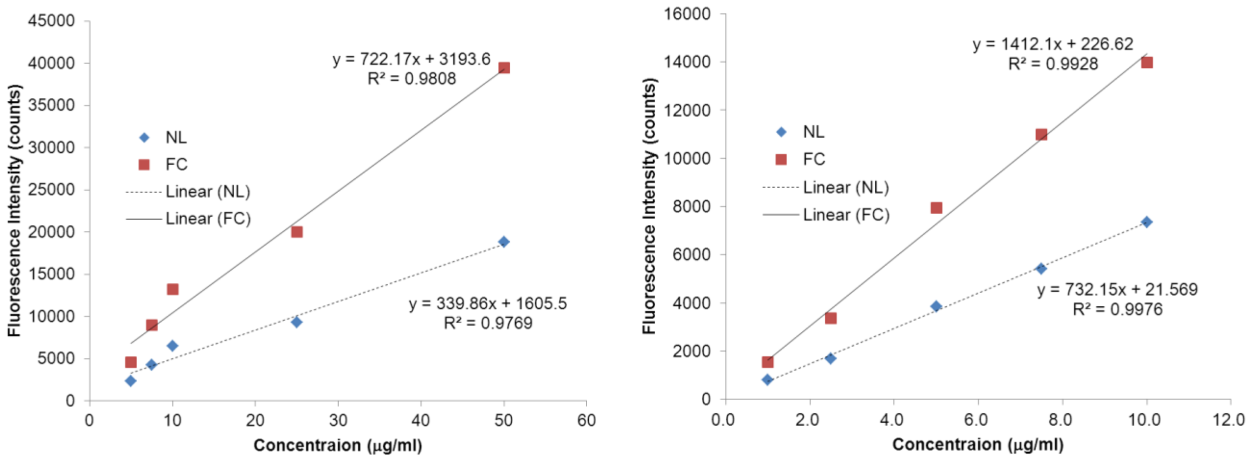

(**a**) (**b**)

Figure S2. The sensitivity (slope) of the fluorescence detections with hybrid lens (HL) and without lens (NL) for (**a**) Nile red and (**b**) Rhodamine 6G. The excitation laser has an incident angle of 15 °.

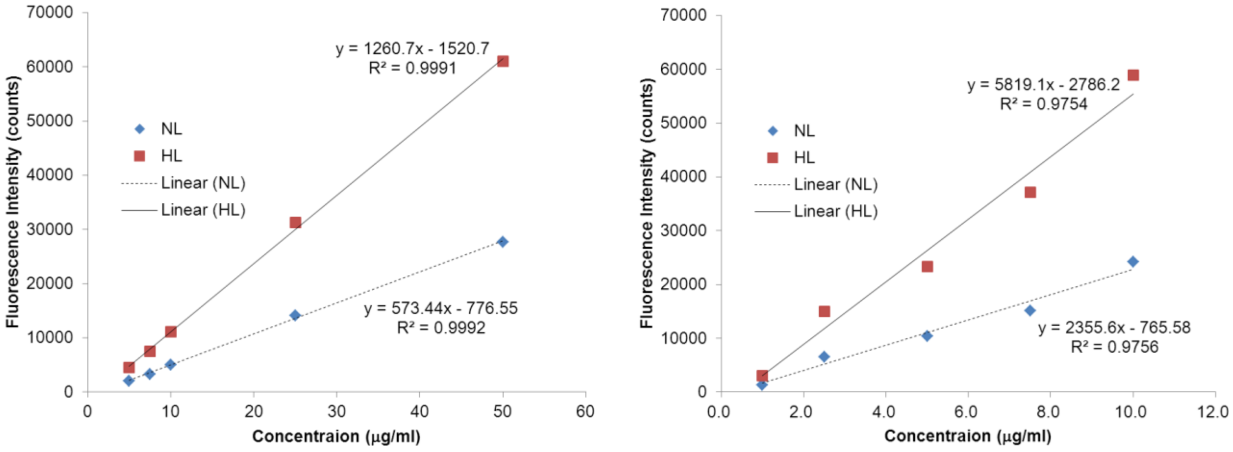

(**a**) (**b**)

Acknowledgments

The authors thank the financial support by Taiwan's National Science Council via grant NSC102-2311-B-006-003-MY3 and NSC102-2221-E-009-065. The support from NCKU's top university grants is also appreciated.

Author Contributions

Bor-Shyh Lin and Han-Yu Yang designed the hybrid lens and performed the simulations of optical paths for the proposed lens. Yu-Ching Yang manufactured the hybrid lens, setup the optical platform, and performed the fluorescence intensity measurements. Chong-Yi Ho performed pretreatments of the samples (dye solutions and *Chlorella vulgaris* cell suspension) for the validation of lens performance. Hsiang-Yu Wang detailed the deployment of PDMS lens to the microfluidic systems for fluorescence measurements.

© 2014 by the authors; licensee MDPI, Basel, Switzerland. This article is an open access article distributed under the terms and conditions of the Creative Commons Attribution license (http://creativecommons.org/licenses/by/3.0/).
